# Health Benefits of Bread Fortification: A Systematic Review of Clinical Trials according to the PRISMA Statement

**DOI:** 10.3390/nu15204459

**Published:** 2023-10-20

**Authors:** Urszula Kaim, Zuzanna Sabina Goluch

**Affiliations:** 1 Department of Bioprocess Engineering, Wrocław University of Economics and Business, Komandorska 118/120, 53-345 Wrocław, Poland; 2 Department of Food Technology and Nutrition, Wrocław University of Economics and Business, Komandorska 118/120, 53-345 Wrocław, Poland; zuzanna.goluch@ue.wroc.pl

**Keywords:** fortification, bread, health benefits, sustainable development, sustainable health

## Abstract

Bread is among the most common foods for the world’s population. Therefore, it can be fortified to eliminate deficiencies of nutrients or be a carrier of other substances with a nutritional or physiological effect, bringing health benefits to its consumers, thus impacting sustainable health. This systematic review aimed to analyze clinical studies on the effects of bread fortification on human health. The study followed the PRISMA guidelines for transparency and utilized databases Scopus, Embase, PubMed, and Web of Science to search clinical trials focused on the effects of bread fortification on human health over the entire last decade. The methodological quality of selected studies was assessed using the Jadad scale. As a result, twenty-six studies meet the inclusion criteria. Clinical trials have shown health benefits from consuming bread fortified with vitamins (B_9_, C, D_2_, D_3_), minerals (K, P, Ca, Mg, Fe, Zn, Cr, Se), fiber, proteins, and polyphenolic compounds. Conclusions reveal that mandatory fortification aligns with the sustainable development goals. Thus, collaborative partnerships are essential for successful implementation and accessibility, fostering public health advances and progress developing into sustainable health.

## 1. Introduction

Fortification is the process of the deliberate addition of nutrients or non-nutrient bioactive components to food products. It is commonly used worldwide to balance nutrition or prevent nutrient intake shortfalls and associated deficiencies. Fortification can also be conducted to provide a sustainable nutritional profile of a diet, to recover nutrients lost during processing, or to offer consumers valuable food products [1]. Fortified foods belong to a group of functional foods that exhibit health-promoting properties that conveniently improve health [2], corresponding with sustainable health assumptions [3].

The term fortification pertains to the incorporation of essential nutrient additives, known as “fortificants”, which, as per the *Codex Alimentarius*, are classified as supplementary constituents or nutrient compounds intended to confer a public health advantage. Food fortification, by definition, is differentiated into mass fortification—for foods widely consumed by the general population; targeted—for foods designed for specific population subgroups (complementary foods for young children or rations for displaced people); and marked-driven—allowing food producers to enrich foods available in the market voluntarily due to customer demands. Mass fortification is practically always mandatory, and a targeted one can be obligatory or voluntary, depending on the public health significance. Market-driven fortification is voluntary and regulated with governed policy for reasonable limits adequate with legislation. Introducing selected fortification types depends on national, political, and geographical circumstances [4].

Food fortification, considered a public health strategy to enhance a population’s nutrient intakes in the past century, has effectively reduced the risk of disorders caused by nutrient deficiency, such as goiter, pellagra, beriberi, or rickets [5]. However, nowadays, the world is challenged with different nutrition-related problems and current fortification programs are designed to increase low dietary intake and prevent malnutrition rather than prevent diagnosable conditions [6]. Bread is one of the most widely embraced food products consumed daily by most people in different countries. Therefore, for years, the food industry has focused on increasing the nutritional value of these products. There are mainly three types of bread produced worldwide: wheat, gluten-free, and mixed. Most bread made from refined flour is poor in vitamins, minerals, fiber, and antioxidants due to the removal of bran and germ in the production, milling, and grinding of grain. 

Furthermore, bread dough needs a thermal process to increase digestibility, palatability, and its physical and nutritional properties, which causes additional reduction or destruction of bioactive compounds. Unfortunately, bread can be poor in nutrients and does not meet consumers’ nutritional and health needs. Therefore, flour is fortified with vitamins and minerals or directly with the bread itself with fiber vegetable extracts, which is essential from a nutritional point of view [7]. Equally significant is consumers’ sensory acceptance of fortified bread, which could diminish due to alterations in taste, aroma, and structure resulting from the inclusion of fortified bread [8,9,10]. 

The nutritional benefits of bread fortification have already been well described in literature reviews [11]. However, to date, no research review has been published on the impact of consuming enriched bread on health benefits. The fortification of staple foods is one strategy with a proven history of improving dietary diversity and effectively decreasing micronutrient deficiencies. Therefore, this systematic review focused on the clinically proven health benefits for the consumer and the resulting directions for further food fortification to maintain good health.

The primary objective of this study is to present a systematic review of clinical trials, with a specific emphasis on bread fortification. Through a rigorous selection of pertinent data, this study evaluates and validates the potential health advantages of consuming fortified bread. Additionally, it seeks to elucidate the role of fortified bread in sustainable health management and accomplishing pre-established goals within the realm of public health.

## 2. Materials and Methods

This review was reported following the Preferred Reporting Items for Systematic Reviews and Meta-Analysis (PRISMA) [12]. A meta-analysis is ineffective in this review because of a lack of homogeneity in selected studies.

The study for this systematic review was restricted to clinical trials carried out in humans in the entire last decade (2013–2022). Four databases were selected for literature research: Ebsco, Pub Med, Scopus, and Web of Science. The search and selection process was performed by two reviewers working independently and in parallel.

**Figure 1:**
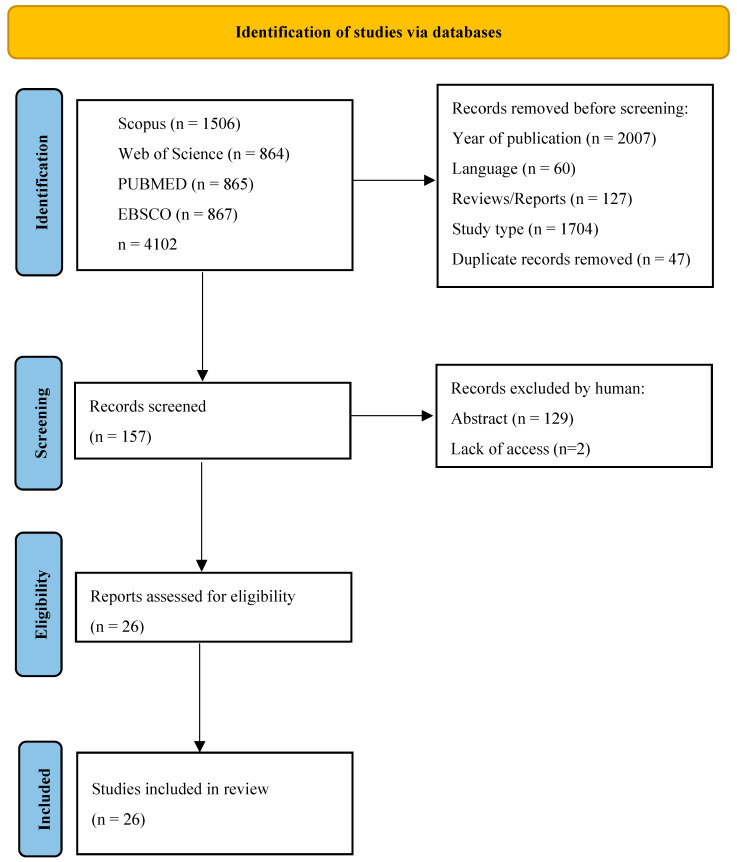
PRISMA flow diagram representing the screening strategy and selection process for research articles. Process of selecting eligible studies.

Searches comprised a combination of MeSH terms and keywords, applying quotes and field tags with Boolean operators. For all databases, five primally exclusion steps were determined: 1. keywords (“bread AND fortifi*”), 2. year, 3. language (English), 4. publication type (article), and 5. study type (clinical trial). For non-medical-only databases, screening was made in the following areas: Title, Abstract, Keywords and Subject Area (Scopus—Core Collection), Topic (Web of Science—Medline), and Ebsco (Health Source, Medline). The last search was carried out on 28 March 2023, due to some publishers’ delay in publishing articles from 2022. Results from each database were exported to files with the appropriate extension of CSV or Excel, and summaries were created for each database containing publication information, including abstracts. The results files are included in Data Availability Statement section The initial search resulted in 4102, from which 2007 was removed because of the year and 1705 because of the study type. After removing non-English publications and reviews and deleting duplicates, 157 records were selected for abstract review. Among these, 129 studies were excluded according to the exclusion criteria, and the full text of 26 eligible studies was included. Only studies involving humans in which comparable parameters were measured before and after the nutritional intervention, such as tests from blood, urine, or feces, were included in the review. Excluding articles occurred for different reasons: technological papers or subject or other simulation studies. Three exclusion criteria were distributed among the rejected studies, such as deviation from the topic: 51%, simulation studies such as digestion or computer simulations: 29%, and others referring to food technology or consumer acceptability. Two independent researchers reviewed the abstracts, and papers that did not meet the criteria for inclusion in the review were excluded through discussion The process of the literature search [12] is presented in Figure 1. 

The Jadad scale was used to achieve high-quality assessment and enhance the validity of research findings [13] in selected clinical trials. The Jadad scale was evaluated with the following assumption: 0–2—low-quality clinical trials, 3—medium-quality clinical trials, and 4–5—high-quality clinical trials.

## 3. Results

The 26 results were divided into four subgroups depending on the type of added fortificant and in accordance with the Codex Alimentarius classification of enriching substances [4]. Out of 26 studies, 9 concerned mineral fortification (Table 1), 4 concerned vitamins (Table 2), 7 concerned nutrients (Table 3), and 6 with physiologically active ingredients (Table 4). 

Among the records, 12 publications were evaluated as low-quality clinical trials, 8 were scored as medium-value trials, and the highest-quality clinical trials were found in 6 papers.

### 3.1. Influence of Fortified Bread on Human Health

#### 3.1.1. Minerals

The effect of fortification of bread with minerals is presented in Table 1. 

El Khoury et al. demonstrated the influence of bread fortification with potassium (K), magnesium (Mg), and phosphorus (P) on human health outcomes. Volunteers were given white pita bread (control) and pita bread fortified with different mineral doses. After 12 h of fasting, volunteers’ blood samples were taken, and they were given 90 g of the tested pita bread (containing 50 g of carbohydrates, and P, K, and Mg, respectively, at 11.6; 12.2; and 2.7 g/kg) with 200 cm^3^ of water. Vein blood samples were collected at 15, 30, 45, 60, 90, and 120 min after meal consumption. Tests with each bread variant were conducted 10 days apart. Glucose, insulin, triacylglycerols, and total P, Mg, and K were determined in the obtained blood serum. Baseline levels of glucose, insulin, homeostasis model assessment of insulin resistance (HOMA-IR), TG, and fasting serum total P, K, and Mg were within normal ranges and were found to be similar between the different experimental sessions for each type of bread. However, it was found that enrichment of white wheat flour with macronutrients (P, Mg, and K) had an effect on lowering postprandial glucose and TG concentrations. The study suggests that fortification of prefilled bread with P, K, and Mg may be a way to improve glycemic response [14]. 

Direct fortification of bread and bakery products with iron (Fe) was presented in three of the included trials [15,16,17,18], one in compilation with zinc [17] and one as undirect fortification with the use of Fe-rich raw material [19]. Fe is the most challenging food fortificant, providing optimal bioabsorption because water-soluble Fe compounds often cause unacceptable color and flavor changes in the food matrix. Moreover, insoluble compounds like elemental Fe powders do not provide sensory changes but are generally characterized by low bioabsorption. Selecting a suitable Fe compound is only a part of the problem. The other difficulty is the presence of its inhibitors in natural food products, such as phytic acid, present in cereal/pseudocereal grains and vegetable seeds or polyphenols in beverages often consumed with meals. The presence of inhibitors can decrease Fe absorption even in its most bioavailable forms [20].

The validity of the first boundary was assessed using the fortification level proposed to be mediatorial in Herter-Aeberli et al.’s trial. The prevalence of anemia is high in Haiti, with 62% of children and 37% of women of a reproductive age. Twenty-two healthy mother–child pairs of preschool age in Haiti received stable Fe isotopes such as FeFum or NaFeEDT in wheat flour bread rolls consumed by all participants in a randomized cross-over design. FeFum + NaFeEDTA was administered in the final identical meal, eaten only by the women. Fe absorption was measured by incorporating stable isotopes into erythrocytes 14 days after each meal, and Fe status, inflammatory markers, and *Helicobacter pylori* infection were determined. In tested groups, fractional Fe absorption (FIA) was considerably higher in meals fortified with NaFeEDTA compared to FeFum. However, FIA from a combination of both additives was not significantly different from the bread with NaFeEDTA. At the same time, the total amount of absorbed Fe was substantially higher for preschoolers when NaFeEDTA was added. All participants were with 40% better absorbed NaFeEDTA compared to FeFum. In considerations, authors suggested optimal amounts of the fortificant in an assumption of a daily intake of 120 g of wheat flour in adults, that the level of fortification should be 30 mg of Fe/kg of flour, and the predicted amount of absorbed Fe would be 0.47 mg/d from NaFeEDTA and 0.33 mg/d from FeFum. Thus, 120 g of such fortified bread would cover 33% of the daily intake of Fe using NaFeEDTA and 24% using FeFum as a fortificant. The authors noted that in Haiti, where the high costs of NaFeEDTA may not be affordable, using FeFum at 60 mg Fe/kg flour may be a preferable, cost-effective fortification strategy [15].

Three studies in these reports corroborate that a potential Fe inhibitor commonly consumed with a meal containing high polyphenol fractions can reduce its absorption [16,17,18]. Results of FIA achieved with assessment of blood samples to measure the incorporation of Fe isotopes in erythrocytes show that iron from NaFeEDTA increases by 17% in the anemia-free group (9.2–24.2%) and 33% in the iron-deficient group (24.2–39.8%) after consumption of bread made from wheat flour fortified with NaFeEDTA. However, rich-in-polyphenol-inhibitors tea consumed within fortified bread drastically reduces FIA (by 85%), showing that NaFeEDTA used as an Fe fortifier cannot overcome the inhibition of tea polyphenols on iron absorption, even in the anemia group, where iron absorption is strongly increased. These results may be valuable for a national Fe fortification strategy [16]. 

In Ndiaye et al.’s trial, Senegalese mother–child pairs were randomly assigned to receive, in a 2 × 2 factorial design, bread fortified with ^58^FeSO_4_ or ^57^FeFum consumed with water or herbal tea of *Combretum micranthum* (Tisane Kinkéliba, TK). The mean fractional iron absorption (FAFe/FIA) was assessed by measuring erythrocyte incorporation of stable iron isotopes 14 days after administration. In the results, FAFe from both breads was comparable, but the mean FAFe from fortified-with-FeSO_4_ bread was higher than for FeFum in children but not mothers. Introducing polyphenol-rich herbal tea additives into tested meals reduced FAFe in mothers by 55.7% and 50.1% from FeSO_4_ and FeFum, respectively, and similar observations were made in children. FAFe decreased by 65.3% from bread fortified with ^58^FeSO_4_ and 72.4% (both *p* < 0.0001) in meals with ^57^FeFum, consuming with a polyphenol inhibitor. Moreover, these results showed that Fe absorption from FeFum is significantly lower than FeSO_4_ in children but not adults [17]. 

**Table 1:** Characteristics of clinical trials in humans regarding the introduction of mineral-fortified bread into their diet (*n* = 9).

Jadad Scale	Participants	Fortificant/Substance/ Dose/Inhibitor	Duration	Methods/Analysis	Results	References/ Country
1	Young male adults; N = 11; Age: 18–30 BMI: 23.8–28.6	Potassium, Magnesium, Phosphorus; 3.6 g of MgCO_3_ and 12.5 g of KH_2_PO_4_/kg of flour; 7.2 g of MgCO_3_ and 25 g of KH_2_PO_4_/kg of flour	30 days	Venous blood; PG; GR; IR; triacylglycerols; total P, Mg, and K; HOMA-IR	TG and GR after consumption of fortified bread maintained at a lower level compared to control.	[14] Lebanon
3	Mother–child pairs; N = 22; Age: 18–45, 2.5–5.	Iron; 2/4 mg of ^57^Fe as FeFum, 2/4 mg of NaFeEDTA (Na58FeEDTA and Na^57^FeEDTA); 2–4 mg/day (4 times during experiment)	28 days	Venous blood, FIA, BIS	FIA from NaFeEDTA is 40% higher than from FeFum; combination of both fortificants does not lead to a significant increase in FIA.	[15] Haiti
1	Women; N = 46; Age: 18–49; Anemic: Hb < 12 g/dL, Non-anemic: Hb ≥ 12 g/dL; BMI: 20.4–25.4	Iron; 6 mg of ^57^Fe as NaFeEDTA, 6 mg (3 mg/3 mg) of ^58^Fe/^56^Fe as NaFeEDTA; Tea	17 days	Venous blood, FIA	Fe from NaFeEDTA increases by 17% in the anemia-free group and 33% in the iron-deficient group; tea consumption reduced iron absorption (>85%) in both groups. NaFeEDTA cannot overcome the inhibition of tea polyphenols.	[16] Morocco
1	Mother–child pairs; N = 17; Age: 18–40, 2–6 BMI: 21.7–22.11.	Iron; 2/4 mg of ^58^FeSO_4_, 2/4 mg of ^57^FeFum; Tea	30 days	Venous blood, FAFe	Fe absorbed from FeSO_4_ was 8.85% higher than for FeFum in mothers, and 56.36% in children. Tea reduced FAFe by 44–65%.	[17] Senegal
0	Women; N = 12; Age: 33–42 BMI: 25.8–30.1	Iron, Zinc; ^58^FeSO_4_, ZnSO_4_; Tea	28 days	Venous blood, CI	Fe absorption from the bread fortified with iron was 19.8% (10.5–37.2%); for 30 mg of Zn/kg, it was 18.5% (10.2–33.4%); 17.7% (7.7–38.7%) for 60 mg of Zn/kg; and 11.2% (6.2–20.3%) for 90 mg of Zn/kg.	[18] Chile
3	Children; N = 82; Age: 2–5.	Iron, Processed amaranth	6 months	Venous blood, hemoglobin, serum ferritin, CCR	Hemoglobin concentration in the amaranth group increased significantly from 112.1 to 120.3 compared to the control (106.9–113.2 g/L); anemia prevalence: CCR (CI:95%) decrease from 0.57 to 0.37 in amaranth group (μU/mL).	[19] Ethiopia
0	Healthy adults; N= 12; Age: 19.6–27.2 BMI: 19.9–24.7	Chromium; Cr-enriched yeast 500 μg of Cr/50 g of available carbohydrates	4 weeks	Venous blood, PG, IR, GR, GI	GR after fortified bread was 101.27 mg/dL and 117.82 mg/dL, compared to control (128.46 mg/dL); GI of enriched breads was 34.22 and 62.35 while the control bread had GI = 100.	[21] Greece
3	Type 2 diabetes adults; N= 30; Age: 40–65 BMI: 27.9–31.1	Chromium; Cr-enriched yeast 99.0 μg of Cr/28 g of bread	12 weeks	Venous blood, BMI, GR, IR	Reduction in BMI from 29.3 to 28.9; decreased fasting plasma glucose from 143.2 to 129.6 (mg/dL); decrease of the mean glycosylated hemoglobin from 6.9 to 6.3 (%); decrease in fasting insulin from 6.8 to 4.4 (μU/mL).	[22] Greece
0	Healthy young adults; Type 2 diabetes adults; N= 10, 10; Age: 23–26, 43–65 BMI: 20–26.57	Chromium, Magnesium, Selenium; Wheat varieties; Cr: 14.0–15.2 μg/100 g Mg: 32.9–85.6 mg/100 g Se: 1.6–5.5 μg/100 g Buckwheat; Mg: 172 mg/100 g Se: 3.5 μg/100 g	2 h	Vein blood, GI, PG	Lower GI of Jizi439 by 19.67% and 10.78% (that of the mixture of white wheat and Chu20); the lowest PG in diabetes patients after Jizi439.	[23] China

Jadad scale—0–5, N—number of population, Age—years, BMI—body mass index (kg/m^2^), HOMA-IR–homeostasis model assessment of insulin resistance, PG—plasma glucose, P—phosphorus, Mg—magnesium, K—potassium, TG—total glucose, GR—glucose response, IR—insulin response, Fe—iron, FeFum—ferrous fumarate, NaFeEDTA—ethylenediaminetetraacetic acid ferric sodium salt, FIA/FAFe—fractional iron absorption, CI—circulating iron, GI—glycemic index, GR—glycemic response, BIS—body iron stores, Hb—hemoglobin, FIA/FAFe—fractional iron absorption, Zn—zinc, CCR—crude relative risk, CI—confidence interval, Cr—chromium.

The evaluation of the effect of Fe absorption in the presence of zinc (Zn) and tea was described in Olivares et al.’s trial. In the study, French-type wheat buns enriched with FeSO_4_ were offered to female participants in approx. 100 g portions consumed with 200 cm^3^ of black tea. On 4 different days and after an overnight fast, the participants were given buns enriched with Fe alone or co-fortified with varying levels of ZnSO_4_ from 30 mg/kg to 90 mg/kg. Radioisotopes with high specific activity (^59^Fe and ^55^Fe) measured by incorporating radioactive iron into erythrocytes were used as markers of iron absorption. The results indicate that the two variants of fortification with zinc levels of 30 and 60 mg/kg did not inhibit Fe absorption. However, Fe absorption significantly decreased at a Zn presence (≥60 mg/kg) when bread was consumed with black tea [18].

In one of the clinical trials, bread was fortified in Fe by using its natural source of a plant origin to determine the possibility of its use in preventing anemia in children. Two-to-five-year-old children without chronic illnesses and with hemoglobin levels from 70 to <110 g/L were included in the study. The intervention and the control group received 150 g of bread daily for 6 months. The intervention–amaranth group was supplied with bread containing 70% amaranth grain and 30% mass of mashed chickpea. The control–maize group was provided with the same portion of bread containing 100% maize. Amaranth grains were processed to reduce phytate availability. At the baseline, 29% of children were diagnosed with anemia, and 71% were mildly anemic. Children with moderate anemia were 38% in the interventional group and 20% in the control group. Fe deficiency constituted 30% of the total anemia cases and was distributed as 35% in the amaranth group and 24% in the maize group. Seven-day food frequency recall shows that 59% of children had not consumed Fe-rich food at least once for 6 consecutive months. There were no significant differences between groups on Fe-rich food consumption. After 6 months, anemia prevalence decreased by 44% compared to 100% at baseline. Children consuming bread enriched with processed amaranth grains showed a 61% reduced risk of anemia and had significantly higher hemoglobin concentrations than the control group [19].

The impact of consuming bread fortified with chromium (Cr)-enriched yeasts on health benefits was analyzed in two included studies. One study was conducted in general healthy young adults [21] and the other in patients with type 2 diabetes [22].

In the first included study, generally, healthy participants were provided with white bread (WB, reference food), whole wheat bread with Cr-enriched yeast (WWCrB, rich in insoluble fiber), white wheat bread with Cr-enriched yeast (WCrB, poor in fiber), or whole-wheat–rye–barley bread enriched with oat beta glucans (BGB, rich in soluble fiber) with 1-week intervals. Of the assayed bread variants, each furnished 50 g of readily available carbohydrates. Blood samples for tests were taken before meals compounded with 100 to 166 g of fortified bread and 250 cm^3^ of water. Subsequent blood samples were taken at 15, 30, 45, 60, 90, 120, and 180 min postprandially. In the results, the glucose response after 180 min of consumption of fortified bread showed significantly lower values, by 8–21% compared to the control, depending on bread variant. The results indicate that consumption of Cr-enriched whole grain bread caused a milder glycemic response and resulted in a significantly lower glycemic index (GI) value than the control bread. The enriched bread had GI values of 38–66% lower than the control. The white wheat bread supplemented with Cr-enriched yeast exhibited the most favorable outcomes regarding postprandial glucose response. Those differences between the bread variants are related fiber content, which may inhibit Cr absorption [21].

The second clinical trial concerning Cr fortification included diabetes participants with a fasting glucose level (FPG) > 125 mg/dL and glycosylated hemoglobin (HBA1c) < 8.5% for 3 months before the study. Disease-related biochemical parameters, inflammatory markers, body weight, and energy balance were studied. After dietary education, groups of participants were given whole wheat bread with Cr-enriched yeast or a placebo in the same daily amount of 112 g of slices of bread. At the end of the experiment, the group consuming fortified bread had lower body weight and lower BMI and decreased FPG, fasting insulin levels, and mean HbA1c. Moreover, the concentration of total Cr in the blood remained within optimal values with a median of 2.1 μg/L, comparable with the values shown in the control group. The study’s results indicate that including Cr-enriched bread in the daily diet of patients with type 2 diabetes can improve glucose tolerance and insulin resistance. Furthermore, it results in a significant reduction in body weight and a significant reduction in systolic blood pressure. No differences were found in oxidative stress and inflammation-related parameters [22].

The effect of consuming micronutrient-enriched wheat steamed bread made from a specific wheat variety, Jizi439, on postprandial plasma glucose levels in both healthy subjects and individuals with type 2 diabetes was investigated by Su-Que et al. [23]. The study included both healthy and diabetic participants. The healthy subjects’ group consisted of young adults, males and females, with standard glucose tolerance and BMI and no family history of type 2 diabetes. The second group consisted of 10 middle-aged and type 2 diabetic patients. The healthy subjects were provided with steamed bread made from Jizi439, Chu20, and a mixture of white grain wheat. Their blood samples were taken at different time points after the meal to measure plasma glucose levels. Diabetic patients consumed bread made from Jizi439, a mixture of white grain bread, wheat, and buckwheat. Blood samples were taken 2 h after the meal to measure plasma glucose levels. The results showed that the consumption of Jizi439 led to the lowest increase in postprandial plasma glucose levels in both healthy and diabetic participants compared to other test foods. Jizi439 had a lower GI and iAUC compared to the other varieties [23].

#### 3.1.2. Vitamins

The effect of fortification of bread with vitamins is presented in Table 2. 

Included studies investigated the impact of folic acid [24], vitamin D_2_ [25], vitamin D_3_ [26], and L-ascorbic acid [27] on the blood glucose response and glycemic index [24], blood folate levels [22], and vitamin D bioavailability [25,26] fortification in bread on various health aspects. 

A study aimed to evaluate the effectiveness of folic acid fortification of wheat rolls and was the first study to use microencapsulated calcium L-5-methyltetrahydrofolic acid as a food fortifier, which should not mask a B_12_ deficiency and may be safer than folic acid. Its influence and an equivalent of folic acid on blood folate levels in study participants was detected. The results were compared to wheat rolls without folate. Both sexes of participants were assigned to three groups and received a wheat roll containing L-5-MTHF, folic acid, or a placebo. After an overnight fast, participants’ blood samples were taken at weeks 8 and 16. In the results, the mean erythrocyte folate was significantly higher in subjects who consumed fortified bread compared to baseline results. The plasma folate was approx. 23 nmol/L higher in the L-5-MTHF and folic acid wheat roll groups than in the placebo group. However, there were no significant differences in blood folate levels between the groups consuming the fortified bread. Results showed that fortified-with-microencapsulated-L-5-MTHF wheat rolls increased erythrocyte and plasma folate concentrations relative to a placebo bread in a healthy population [24].

**Table 2:** Characteristics of clinical trials in humans regarding the introduction of vitamin-fortified bread into their diet (*n* = 4).

Jadad Scale	Participants	Fortificant/Substance/ Dose/Inhibitor	Duration	Methods/Analysis	Results	References/ Country
4	Adults N = 45 Age: 18–45 BMI: 18.5–30	Folic acid; Folate, Calcium L-5-methyltetrahydrofolic acid; 452 mg of L-5-MTHF/roll; 400 mg of folic acid/roll	16 weeks	Venous blood, Plasma folate, Whole-blood folate concentrations	Increase in mean erythrocyte folate by 60% for L-5-MTHF, and by 44% for folic acid.	[24] Canada
3	Adult women N = 41 Age: 20–40 BMI: 19.9–24.5	Vitamin D_2_, UV-treated baker’s yeast; 30.2 μg/100 g	8 weeks	Venous blood sample, Serum 25-hydroxyvitamin D concentrations	Increase in S-25(OH)D_2_ concentration by 6.4 nmol/L.	[25] Finland
4	Adults N = 90 Age: 26–48 BMI: 21.7–31.2	Vitamin D_3_, Vitamin premix, 25 μg/50 g		8 weeks	Venous blood sample, serum 25-hydroxyvitamin D concentrations.	[26] Iran
0	Young adults N = 27 Age: 20–29 BMI: 20–24	Vitamin C; L-ascorbic acid; 5 ppm, 10 ppm, 15 ppm	120 min (30, 60, 90, and 120 min)	Venous blood, GR, GI, GL	GR was the lowest for 5 ppm ascorbic acid brad: 85, 98, 101, 93, 92 for 72% flour extract, and 91, 90, 95, 91, 86 for 72% flour extract; lowest GI (83–90% flour extract) in variant fortified with 10 ppm ascorbic acid, lowest GL (42–90% flour extract) in variant fortified with 10 ppm ascorbic acid.	[27] Iraq

Jadad scale—0–5, N—number of population, Age—years, BMI—body mass index (kg/m^2^), GR—glucose response, GI—glycemic index, GL—glycemic load, L-5-MTHF—calcium L-5-methyltetrahydrofolic acid, 25(OH)D—serum 25-hydroxy vitamin D.

Investigation of potential benefits related to the consumption of bread fortified with D_2_-fortified yeast to prevent vitamin deficiency was conducted in endogenous subjects in late winter/early spring when UVB sunlight was insufficient. Female participants were divided into four study groups and received regular wheat bread with a placebo pill, a daily D_2_ supplement, a D_3_ supplement, or D_2_-biofortified wheat bread with a placebo pill. The average daily serving of bread weighed 87 g. The average increase in the D_2_ supplement group was 9.6 nmol/L (+14.7%); for the D3 supplement group, it was 17.0 nmol/L (+26.2%). D_2_-enriched bread groups showed a modest increase in the total S-25(OH)D, while it remained stable regarding the placebo. The study found that the D_2_ form used to fortify the bread did not have good bioavailability to humans, probably due to conditions during the baking process or its potentially indigestible form [25]. 

A study by Nikooyeh et al. detected the bioavailability of vitamin D from fortified bread and the effects of its daily consumption on health aspects. A premix containing 100,000 IU/g of flour with vitamin D_3_ was added to the Lavash bread formulation to provide a dose comparable to a 1000-IU vitamin D supplement. Both sexes of participants were assigned into three groups consuming 50 g of bread fortified with 25 g of vitamin D_3_ and a placebo supplement daily; another group consumed plain bread and 25 g of a vitamin D_3_ supplement; the control group consumed plain bread and a placebo pill. After 8 weeks of intervention, the within-group change in serum 25(OH)D concentrations was significantly higher in the enriched-bread-eating group than in the supplement group and placebo group. Results indicated that vitamin-D-fortified bread could effectively raise the population’s circulating 5-hydroxyvitamin D levels to nearly optimal. There were no significant differences in laboratory and anthropometric parameters between the study groups [26].

The effect of L-ascorbic acid addition to Iraqi flatbread on the blood glucose response and glycemic index was detected by Thannoun and Al-Arajy. The study was conducted with generally healthy young adults divided into three groups who received two types of fortified wheat bread (72% or 90% flour extract) with different concentrations of ascorbic acid. Blood glucose response was determined at 0, 30, 60, 90, and 120 min after the meal. Bread fortification with ascorbic acid resulted in a reduction in the glycemic index and glycemic load. These findings were more significant in bread made from flour with 90% extraction than 72%, probably due to the higher amount of dietary fiber. The glucose response was the weakest after eating bread enriched with 5 ppm ascorbic acid, made from the 72% flour extract. However, the glycemic index and load have the lowest values in the bread made with the 90% flour extract in the variant fortified with 10 ppm ascorbic acid and were lower than in whole grain bread. The study shows that the analyzed parameters are more influenced by the degree of flour milling than by the amount of fortificant added, as the results of the measured parameters did not differ significantly [27]. 

#### 3.1.3. Other Nutritional Compounds

The clinical trials on nutrient enrichment (Table 3) of bread included in this review involved fiber [28,29,30,31], protein [32], and plant-based substances containing high levels of both protein and fiber [33]. Four were conducted with patients who have second-degree diabetes [32,33] and two with obesity [32,34]. 

##### Fiber

The influence of fiber fortification of bread with vegetable powder on serum glucose levels, insulin response, and subjective appetite suppression was investigated by Amoah et al. The bread was fortified with pumpkin and sweet corn powder. Participants were tested three times after an overnight fast and consumed 75 g of vegetable-enriched bread, with pumpkin powder or sweet corn powder, or white control bread. The vegetable-enriched bread was formulated from the following ingredients: strong white wheat flour, wholemeal wheat flour, whole flaxseed, sprouted wheat flour, pumpkin flour, sweet corn flour, fresh yeast, and salt (descending order by weight). Participants assessed their subjective sensations of hunger, fullness, satisfaction, and desire to eat using a Likert scale. Venous blood samples were taken at 0, 15, 30, 45, 60, 90, and 120 min after bread consumption and were analyzed for glucose and insulin levels. After 120 min of consumption, serum glucose levels were 19.1 mmol*min/L lower for enriched bread compared to the control. Moreover, the mean AUC of insulin for the fortified bread was significantly lower, with a difference of 12.415 pmol/L*min. In addition, consuming enriched bread was associated with greater feelings of fullness in participants over 120 min due to the presence of hydrophilic fiber pectin, cellulose, and β-glucan; flaxseed and whole grain flour suppressed postprandial appetite and improved the glycemic response; and sprouted wheat lowered the glycemic index [28].

Boll et al.’s trial investigated the metabolic benefits of adding probiotic arabinoxylan oligosaccharides and resistant starch to white wheat bread on overnight glucose tolerance, intestinal fermentation, and changes in gut-derived hormones. The study involved healthy young adults consuming three test products or control bread in equal carbohydrate portions of 50 g of available starch. Breads were consumed late at night with 250–300 cm^3^ of water. The fortification was carried out using a wheat bran extract rich in arabinoxylan oligosaccharides, resistant corn starch, or a combination of both. The amount of additives in the bread recipe was 120 g of starch (Hi-Maize™ 260) and 90 g of the wheat bran extract (Brana Vita™ 200) or 165 g of the same wheat bran extract or 270 g of high-amylose corn starch in the mixed bread. On four occasions, fasting blood samples were collected to measure blood glucose, serum insulin, plasma GLP-1, H2 respiration, and subjective appetite sensation that were monitored before breakfast and at 15, 30, 45, 60, 90, 120, and 180 min afterwards. The overall postprandial fasting glucose response was not statistically different for the bread variants. However, a significant dose-dependent decrease in glucose response was observed with increased wheat bran extract content for fortified breads. A similar observation was made for fasting insulin. With a higher dose of the wheat bran extract, insulin levels decreased in fortified breads. The amount of high-resistivity corn starch did not cause significant decreases. In addition, the insulin sensitivity index improved with a similar trend after consuming bread enriched with the compilation of additives [29]. 

Stewart et al. aimed to assess postprandial glucose, insulin responses, postprandial satiety, and gastrointestinal tolerance after consuming wheat scones with high dietary fiber content and resistant starch (RS). The bread was fortified with VERSAFIBE™2470 resistance starch containing 70% dietary fiber from high-amylose corn starch. Healthy subjects with fasting blood glucose above 5.55 mmol/L participated in three trials, and baseline satiety was assessed 30 min before consuming the bread with 240 cm^3^ of water and 15 min after the first blood sample was taken, followed by 180 min to measure blood glucose and insulin concentrations. Moreover, respondents completed the Satiety Visual Analog Scales (VAS), Gastrointestinal (GI) Tolerability Questionnaire, and palatability questionnaire. The results showed that consuming the fiber-enriched scones significantly reduced postprandial glucose and insulin incremental areas under the curves (43–45% reduction and 35–40% reduction, respectively) and postprandial glucose and insulin maximum concentrations (8–10% and 22% reduction, respectively). The fiber scone significantly reduced hunger and desire to eat during the 180 min following consumption. In addition, no gastrointestinal side effects were observed [30].

A study of the effect of arabinoxylan-fortified white bread on postprandial glucose levels was conducted by Guilia Falchi et al. Healthy adult volunteers were asked to consume 100 g of white bread containing refined wheat flour after a 4-h fast or the same amount of bread enriched with arabinoxylan (3.2%). No significant differences were observed by the study subjects between the two bread formulations in terms of flavor, color, texture, and overall quality. Blood glucose was assessed using a glucometer just before the bread was consumed and after 30 min. It was observed that the postprandial glucose concentration after consumption of enriched bread was significantly lower than (by 20%) that of the control with values of 107 ± 4.6 mg/dL vs. 121 ± 5.2 mg/dL [31].

##### Protein

Investigating the effect of adding whey protein to bread in overweight or obese women with type 2 diabetes on the glycemic status, lipid profile, and blood pressure was the aim of a 12-week study by Nouri et al. In the trial, patients were assigned to a group consuming bread fortified with 20 g of a whey protein concentrate or to a placebo group. At baseline, parameters such as glucose, serum insulin, lipid profile, blood pressure, glycosylated hemoglobin HBA1C, physical activity, and dietary intake were determined. Insulin resistance was determined by evaluating the homeostatic model for insulin resistance HOMA-IR. In the results, except for the HbA1C value, which was higher in the experimental group, there were no significant differences in glycemic parameters. In addition, fasting glucose levels were significantly elevated in the experimental group. Insulin levels in both groups also increased. However, no significant differences were observed between the groups in the participants’ lipid profiles and blood pressure [32].

The study conducted by Moghaddam et al. aimed to assess the impact of bread fortified with soybean flour on the metabolic profile of women with type 2 diabetes. Initially, a 2-week run-in period was implemented, followed by random assignment of participants to either the intervention or control group. During the intervention phase, participants in the intervention group were instructed to replace an equivalent portion (120 g) of their regular bread or cereal intake with soybean-flour-fortified bread for 6 weeks. After a 4-week washout, the participants crossed to the alternative regimen for another 6 weeks. The study results revealed no statistically significant effects of soybean-flour-fortified bread on the metabolic profile of participants. While specific metabolic parameters, such as serum triglycerides, serum low-density lipoprotein cholesterol, insulin, and homeostatic model assessment of insulin resistance, decreased after 6 weeks, these changes did not reach statistical significance. Additionally, no significant alterations were observed in anthropometric indices, blood pressure, fasting blood sugar, glycated hemoglobin, high-density lipoproteins, and total cholesterol levels after consuming soybean-flour-fortified bread [33].

Sharifi-Zahabi et al. [34] conducted a study among overweight and obese women to investigate the effects of consumption of bread fortified with soy flour on factors influencing cardiovascular disease (CVD) risk. The study consisted of two diet phases: a treatment diet (containing 120 g of soy bread) and a control diet (a regular diet with a calorie deficit). The soy bread was formulated by substituting 30% of the wheat flour with roasted soy flour. Each diet lasted 6 weeks, with a 20-day washout period between the two phases. Measurements were taken at baseline and after 6, 9, and 15 weeks. Blood samples were collected after an overnight fast to measure the lipid profile and other variables. The study found no significant effects of soy bread on serum lipid levels, systolic blood pressure, and anthropometric indices compared to the regular diet. However, soy bread had a marginally significant effect on diastolic blood pressure (DBP). Specifically, in the E2 genotype group (ɛ2/ɛ2), soy bread significantly affected DBP compared to the regular diet. Results suggest that individuals with the ɛ2 allele may respond differently to soy bread consumption regarding CVD risk [34].

**Table 3:** Characteristics of clinical trials in humans on introducing bread fortified with various ingredients with a nutritional effect into the diet (*n* = 7).

Jadad Scale	Participants	Fortificant/Substance/ Dose/Inhibitor	Duration	Methods/Analysis	Results	References/ Country
0	Young adults N = 10 Age: 16–30 BMI: 27.6–36.6	Fiber; pumpkin powder at 75 g/450 g of flour, corn powder at 20 g/450 g of flour	3 days	Vein blood, SG, IR, SAS	SG after 120 min: enriched bread at 56.8 L vs. 75.9 mmol*min/L for control bread; MI after 120 min: 20,476 vs. 32,892 mmol*min/L; lower SAS	[28] New Zealand
2	Young adults N = 19 Age: 22–24 BMI: 22.2	Fiber; arabinoxylan oligosaccharides: 8.9 g/portion, 18.4 g/portion; resistant starch: 6.6 g/portion, 15 g/portion	3 h	Vein blood, GR, IR, ISI	GR decrease from 183.3 mmol min/L to 168.3 to 154.4 mmol min/L at 0–120 min; IR decreased from 0.050 nmol/l to 0.042 and 0.038 nmol/l; ISI increase from 478 to 512 and 551	[29] Sweden
5	Adults N = 35 Age: 17–74 BMI: 18.5–29.99	Fiber; VERSAFIBE™2470 at 16.5 g/portion	3 weeks	Vein blood, GR, IR, PS, gastrointestinal tolerance	GR reduced by 43–45%, IR by 35–40%, reduced hunger	[30] USA
4	Adults N = 24 Age: 34–61 BMI: 22.1	Fiber; arabinoxylan at 3.2 g/100 g	7 days	Vein blood, PPG	PPG reduced by 20%	[31] Italy
3	Overweight or obese women with type 2 diabetes N = 48 Age: 25–55 BMI: 25–40	Protein, whey protein at 20 g/portion	12 weeks	Vein blood, GS, FGL, IL, LP, BP	FGL elevated from 154.50 to 178.50 mg/dL; IL increased from 22.45 to 44.30 μIU/mL and from 16.30 to 38.30 μIU/mL in experimental and placebo group, respectively	[32] Iran
3	Diabetes patients, women N = 30 Age: 30–50 BMI: 29.5	Protein, soy flour	8 weeks	Vein blood, metabolic profile (n > 30)	No statistically significant changes	[33] Iran
3	Overweight/obese women N = 30 Age: 19–35 BMI: 25–30	Protein, soy flour	15 weeks	Vein blood, LP, BP, anthropometric parameters, CVD factors, BMI, weight	Weight reduction by 4.8 kg; BMI reduction by 0.6	[34] Iran

Jadad scale—0–5, N—number of population, Age—years, BMI—body mass index (kg/m^2^), SG—serum glucose, IR—insulin response, MI—mean insulin, SAS—subjective appetite suppression, GR—glucose response, IR—insulin response, PPG—postprandial plasma glucose, PS—postprandial satiety, ISI—insulin sensitivity index, GS—glycemic status, FGL—fasting glucose level, IL—insulin level, LP—lipid profile, BP—blood pressure, CVD—cardiovascular disease.

#### 3.1.4. Physiological Compounds

Table 4 shows included studies focused on the effects of the addition of physiological compounds such as phytochemicals [35], polyphenols [36], phenolic acids [37], phytosterols [38], and phytochemicals [39] into bread on different aspects of metabolic health. These aspects included metabolic risk factors [35], their parameters [36] and responses [37], as well as lipid profiles [38] and glycemic and insulin responses [39]. Additionally, one study explored the effects of neurotransmitter and enzyme inhibitors on blood pressure, glucose metabolism, and endothelial function [40]. 

The study of Apprey et al. aimed to assess the impact of *Borassus aethiopum*-fortified bread on metabolic risk factors in outpatients with CVD. Borassus fruit pulp contains phytochemicals, including flavonoids, alkaloids, triterpenes, steroids and sterols (cardiac glycosides), saponins, and phenols, as well as substantial antioxidant levels. The 122 patients with CVD were given either Borassus-fortified bread (150 g) or an indistinguishable placebo (150 g of white flour bread) daily for 90 days. Body composition, blood pressure, and biochemical parameters were measured before and after the intervention. Following the intervention, there was a significant reduction in mean waist circumference, BMI, and visceral fat. Additionally, systolic blood pressure, diastolic blood pressure, serum total cholesterol (TC), LDL, and HDL levels within the experimental group showed significant reductions regarding both before and after measurements. According to the authors, the potential of *Borassus aethiopum* in treating cardiovascular and other metabolic diseases should be examined [35].

The influence of soy bread fortified with plant powders, turmeric (5%), and ginger (5%) on metabolic parameters of obese and type 2 diabetes patients was the focus of the Fouad et al. trial. In the first phase of the experiment, one group of participants followed a low-calorie balanced diet (1000–1200 kcal), consuming traditional Syrian bread fortified with soy flour and 5% turmeric powder in two portions per day, and the second group consumed soy bread fortified with 5% ginger powder and followed the exact instructions. Phase 2 lasted for 4 weeks, during which participants continued the low-calorie diet without consuming any food containing soy products. Study results demonstrated that curcumin exhibited more significant hypolipidemic benefits compared to ginger, while ginger showed superior antidiabetic properties compared to curcumin. However, both curcumin and ginger exhibited antiobesity effects. The inclusion of curcumin in bread resulted in a significant reduction in anthropometric parameters such as BMI, mean body weight, waist circumference, WHtR, and WHR and lipid profiles, including a decrease in total cholesterol, non-HDL-C, LDL, and TG, and an increase in HDL levels. On the other hand, ginger, a natural antidiabetic agent, demonstrated a more significant reduction in the change in insulin sensitivity, fasting blood glucose levels, and hemoglobin HbA1c compared to curcumin [36]. 

The objective of the Lappi et al. [37] study was to investigate the impact of enzyme and yeast bioprocessing of rye bran on the absorption of phenolic acids in healthy individuals. Rye bran contains phenolic acids with potential health benefits; however, their absorption in the human body is limited due to their binding to dietary fiber. 

**Table 4:** Characteristics of clinical trials in humans on the introduction of bread enriched with various physiological ingredients, and its effect, into the diet (*n* = 6).

Jadad Scale	Participants	Fortificant/Substance/ Dose/Inhibitor	Duration	Methods/Analysis	Results	References/ Country
3	Diabetes patients N = 122 Age: 19–50+ BMI: 22.7–38.3	Phytochemicals/polyphenols; Borassus powder: (wheat flour: BRP; m:m) 2:1; 5:1; 10:1; 20:1	3 months	Venous blood, Physical parameters Visceral fat, Metabolic age SBP, DBP, TC	waste reduction from 98.3 ± 14.6 cm to 95.9 ± 15.8 cm; BMI reduction by 3.4; visceral fat reduction from 10.4 ± 3.2 to 9.9 ± 3.0; metabolic age reduced from 61:6 ± 13:4 to 59:3 ± 13:0; SBP reduced from 161.2 ± 25.5 to 137.6 ± 22.9; DBP reduced from 99.2 ± 13.6 to 85.1 ± 10.8; TC reduced from 5.9 to 4.9; LDL reduced from 3.4 to 2.8; HDL reduced from 2.2 to 1.5	[35] Ghana
0	Diabetes patients Women N = 72 Age: 46–51 BMI: 35–37	Polyphenols; Turmeric powder: 10/100 g Ginger powder: 10/100 g	8 weeks	Vein blood, Physical parameters (>10), CVD risk factors	BMI reduction from 37 to 36, 35 to 34; BF reduction of 1–1.5%; TG reduction from 139.28 to 118 mg/dL, 135 to 102 mg/dL; TC from 234 to 205, 218 to 183; CVDrf decreased from 5.17 to 3.99, 4.73–3.63	[36] Egypt
0	Healthy adults N = 15 Age: 35–65 BMI: 21–32	Phenolic acids, Native ray bran: Ferulic acid—134 mg/portion; Sinapic acid—31 mg/portion; 4-Coumaric acid—4.7 mg/portion; Bioprocessed rye bran: Ferulic acid—117 mg/portion; Sinapic acid—28 mg/portion; 4-Coumaric acid—5 mg/portion	24 h	Vein blood, Urine samples; Postprandial phenolic acid excretions, GR	Bioprocessing of rye bran increased FA absorption by 14% vs. native rye bran	[37] Finland
5	Healthy adults N = 36, 39 Age: 18–70 BMI: <40 kg	Phytosterols; Vegapure^®^ 67WDP (BASF): 2.3g/2 slices; Curcumin: 228 mg/2 slices	4 weeks	Venous blood, TC, LDL, CVD risk	TC decrease of 0.52 mmol/L; LDL decrease of 0.49 mmol/L–124.33 nmol/L; CVD risk decreased from 1.1 to 12.7%	[38] Australia
1	Healthy adults N = 13 Age: 20–46 BMI: 19.4–29.7	Polyphenols; Green tea extract powder: 0.4% of loaf weight in g; Baobab fruit extract powder: 1.88% of loaf weight in g	6 days	Vein blood, GR, IR, Satiety	No statistically significant changes	[39] Australia
5	CVD risk patients N = 30 Age: 18–65 BMI: 23–31	Neurotransmitters, Enzyme inhibitors, γ-Aminobutyric acid: 22.8 mg/100 g; Angiotensin-converting enzyme inhibitor: 7.23 mg/g (LSBþG)	112 days	Vein blood, Urine samples, BP, GM, Endothelial function	BP decreased for 2.12 mmHg for LSBþG	[40] Spain

Jadad scale—0–5, N—number of population, Age—years, BMI—body mass index (kg/m^2^), SBP—systolic blood pressure, DBP—diastolic blood pressure, BP—blood pressure, TC—serum total cholesterol, LDL—low-density lipoprotein cholesterol, HDL—high-density lipoprotein cholesterol, TG—triglyceride, GR—glycemic response, IR—insulin response, GM—glucose metabolism, BF—body fat, CVDrf—cardiovascular disease risk factor.

Bioprocessing techniques involving enzymes and yeast were employed to release the phenolic acids from the fiber complex. The study involves subjects consuming different types of bread, such as wheat bread fortified with either bioprocessed or native rye bran and whole grain rye bread or wheat bread as a control. Urine samples were collected at various time points (basal state, and 4, 8, and 12 h) over 24 h to measure phenolic acids and their metabolites. Additionally, blood samples were taken six times over 4 h to assess postprandial glucose and insulin responses following bread ingestion. The results showed that bioprocessing of rye bran increased the proportion of free ferulic acid (FA) and soluble arabinoxylan in the bread. Consumption of white wheat bread fortified with bioprocessed rye bran led to a significant increase in the urinary excretion of FA, particularly within the first 4 h. This finding suggests an enhanced absorption of FA from the small intestine due to bioprocessing. However, the bread types had no significant differences in postprandial glucose and insulin responses. Additionally, the bioprocessing of rye bran did not affect the excretion of benzoic, phenylpropionic, and phenylacetic acid metabolites. In conclusion, compared to native rye bran, the bioprocessing of rye bran increased the absorption of ferulic acid from the small intestine in healthy individuals [37].

The Ferguson et al. study aimed to examine the impact of a combination of ingredients (fortified-with-phytosterol bread) on the lipid profiles of individuals with high cholesterol levels. In a clinical trial employing double-blinding and a placebo, a factorial design was used, with participants randomly assigned to consume bread fortified with phytosterols (PSs) (Vegapure, 67WDP), curcumin, or a combination of both, or a placebo. The primary outcomes assessed were fasting plasma lipid levels, TC, LDL, HDL, and triglycerides (TGs). Secondary outcomes included the profile of LDL particles (LDL-P), including LDL-P number and LDL-P size. Additionally, the study explored the impact of CVD risk using the Framingham Risk Algorithm. No significant differences were observed between interventional groups concerning blood lipid levels or CVD risk. In the PS-C group, there was a significant decrease in TC, LDL-C, and CVD risk. HDL-C and TG levels remained unchanged. There was a significant reduction in LDL-P in the PS-C group compared to the PL-C group. Both groups exhibited a significant decrease in LDL-P size; however, the absolute change in LDL-P size did not differ significantly between the groups, and the percent change in LDL-P size in the PS-C group showed borderline significance (−0.89%) compared to the PL-C group. Regular consumption of PS-enriched bread with or without curcumin lowers blood cholesterol levels in people with hypercholesterolemia [38].

The influence of the incorporation of polyphenol-rich extracts into wheat bread on the glycemic and insulin responses in healthy individuals was detected in the Coe et al. [39] study. To determine the optimal dosage of different extracts (including baobab fruit extract, green tea extract, grape seed extract, and resveratrol) for reducing rapidly digestible starch in white bread, an in vitro dose–response analysis was conducted. Two extracts demonstrating the highest potential for reducing sugar were selected for a trial involving both sexes of volunteers in a crossover study featuring three different meal conditions. On separate occasions, the participants consumed 250 cm^3^ of water and control white bread, white bread fortified with the green tea extract in the amount of 104.16 g, bread with a total of 96.33 mg of added polyphenols, and white bread fortified with the baobab fruit extract in the amount of 106.97 g with 61.24 mg of total added polyphenols. The glycemic response, insulin response, and satiety levels were measured 3 h after the meals. Although the enriched bread did not lead to a significant reduction in glycemic response or hunger, the white bread supplemented with the baobab fruit extract exhibited a noteworthy decrease in the total (0–180 min) and segmental insulin area under the curve at 0 to 90, 0 to 120, and 0 to 150 min. However, there was a nonsignificant trend for fortified bread to have a lower peak in detected parameters than control bread and no differences in satiety [39].

Becerra-Tomas et al. [40] conducted a study to investigate the potential effects of low-sodium bread fortified with potassium, g-aminobutyric acid (GABA), and angiotensin-converting enzyme inhibitor (ACEI) peptides on 24-h blood pressure (BP), glucose metabolism, and endothelial function. Patients with pre-hypertension or mild-to-moderate hypertension participated in the trial, consisting of three 4-week nutritional interventions, each separated by 2-week washout periods. During each intervention, the patients consumed 120 g/day of one of the three types of bread: conventional wheat bread (CB), low-sodium wheat bread enriched in potassium (LSB), and low-sodium wheat bread rich in potassium, GABA, and ACEI peptides (LSB+G). In each period, 24-h BP measurements, in vivo endothelial function, and biochemical samples were obtained. Although not statistically significant, the intervention period with LSB+G showed a tendency towards a higher decrease in 24-h BP compared to the interventions with CB or LSB alone. The mean change in systolic BP was for LSB+G. However, the differences in BP changes between the treatments did not reach statistical significance. There were no significant differences between interventions regarding changes in in vivo endothelial function, glucose metabolism, and peripheral inflammatory parameters [40].

### 3.2. Health Benefits of Consumption of Fortified Bread

Based on the information described in the previous sections of this article, consuming bread fortified with minerals, vitamins, nutrients, and physiological compounds could have significant beneficial implications for human health. 

Chromium fortification in bread can optimize glucose metabolism and mitigate the risk of insulin resistance and type 2 diabetes. Fortification of bread with Cr-enriched yeast presents a new strategy for managing the glycemic response to bread [21,22]. Natural rich-in-Cr wheat varieties could provide medication and nutritional values, which create a source to prevent diabetes and cardiovascular diseases and enhance immunity [23]. Furthermore, fortification with other minerals such as P, Mg, and K may reduce postprandial glucose and TG levels, which supports the benefit of increasing the consumption of whole grain wheat since it retains most of its mineral content. These findings may significantly ameliorate simple carbohydrates’ detrimental potential effect [14]. 

Moreover, the addition of vitamin C to 90% extracted flour (wholemeal) can improve GI, GR, and GL in healthy participants. Vitamin C fortification can provide an additional quencher to enhance the natural processes of glucose metabolism and help reduce the rapid release of post-meal glucose [27]. 

Fortification of bread with vegetable powders and fiber is also associated with a postprandial reduction in postprandial serum glucose and insulin release. Moreover, its consumption increases the feeling of fullness compared with traditional bread [28,31,36]; thus, it can be used in diabetic and obese patient diets. Those results are also achievable by using resistant starch as a fortificant [29] or bioprocessed rye bran [37]. 

Replacement of traditional bread with bread fortified with polyphenol-rich fruit powder results in improvement in the lipid profile in CVD patients with a significant reduction in SBP, DBP, TC, LDL, and HDL [35,36].

Furthermore, fortification of bread with neurotransmitters and enzyme inhibitors reveals a positive effect on the lipid profile in CVD-risk patients by decreasing BP [40]. 

Additional protein in bread fortified with soy flour can decrease the BMI index and weight in overweight and obese patients after 15 weeks of replacement of traditional bread [34]. Fortification of bread with polyphenol-rich powder results in reduction in waste, BMI reduction, and visceral and body fat reduction (what can be used in obesity). Its consumption also influences metabolic age by statistically decreasing it [35,36]. 

Fe fortification of bread effectively addresses Fe deficiency, particularly in populations with inadequate dietary intake of this mineral. Depending on the region, a higher fortification level with the selected fortificant or native raw materials rich in Fe might result in optimal mineral absorption and, therefore, be more cost-effective. However, the strong inhibitory effect of polyphenol-rich beverages commonly consumed with meals reduces the amount of absorbed Fe from the fortified flour. Thus, those drinks should be replaced during meals with beverages with a good source of ascorbic acid to improve the impact of the national iron-fortification program. Because of the above, nutrition education should be conducted to ensure proper nutritional health in countries where tea is often consumed with meals that include products made from refined flour [15,16,17,19]. 

Regarding health benefits, adequate folic acid intake is paramount in improving cardiovascular health by reducing homocysteine levels and potentially lowering the risk of related diseases [24].

Regarding vitamin D, fortifying bread offers a valuable opportunity to prevent bone-related disorders like rickets in children and osteomalacia in adults [25]. Fortification of staple foods, including bread, with vitamin D becomes an effective strategy to ensure sufficient intake, especially among populations at risk of deficiency [26].

Including protein fortification in health policy initiatives can positively impact public health by addressing protein deficiencies and promoting better nutrition for populations. In the realm of physiological compounds, fortification with polyphenols, phytosterols, phenolic acids, and neurotransmitter precursors presents an opportunity to augment nutritional value and improve health outcomes. Fortifying foods with these bioactive compounds can contribute to nutrient enrichment and elevate the nutritional quality of the food supply, addressing nutrient deficiencies and enhancing public health [41]. Moreover, integrating these compounds into health policies can bolster disease prevention initiatives, targeting specific health conditions and reducing the burden of chronic illnesses [42]. Fortification initiatives can promote healthy dietary habits, encouraging consumers to select foods with supplementary health benefits and fostering balanced diets, thus creating a sustainable health approach.

## 4. Discussion

Three incorporated trials disclosed external funding sources, denoted by authors Stewart, Guilia, and Becerra [30,31,40]. Within one of these papers, an author was directly affiliated with the entity providing financial support [30]. However, in two instances, information about potential conflicts of interest was either undisclosed or not provided [18,36]. 

From the analysis of clinical trials on the benefits of bread fortification with various compounds, their limitations emerged, which may have influenced the authors’ conclusions, such as lack of preliminary studies on the bioavailability of bread fortifiers; different number of people participating in the research (from 9 to 122 people); different age of respondents (from 2 years to 75 years); different times of intervention (up to 24 h to 6 months); different doses of fortifiers; assessment of the overall effect of fortifiers, not individual compounds; the use of research in people who did not have drastic deficiencies of fortified ingredients; conducting tests in healthy people or selected diseases (anemia, hypercholesterolemia, diabetes, CVD); not taking into account the ethnicity and gender of participants (only women in some studies); not taking into account the usual diet (assessment of consumption only from 24 h) and nutritional status (only with average weight, overweight, obesity); the use of different portion sizes of bread for daily consumption (up to 160 g); lack of control over participants’ total food intake and physical activity during the intervention period; and no information on the drugs used by the study participants.

Based on the analysis of clinical trials, it seems that in the future it is justified to search for new bread fortifiers, performed by food technologists, especially from raw materials of a natural origin (composition, doses, matrix) and to reduce anti-nutritional substances in order to minimize the possible adverse effect on the organism; conduct research on the bioavailability of fortificants, taking into account age, gender, ethnicity, and various disease entities; assess whether single-component or multi-component fortification is more effective in terms of health (and in what proportions); place, on the packaging of fortified bread, information on with which other foodstuffs/dietary supplements/medicines it should not be eaten, which is due to possible interactions leading to changes in bioavailability; and consider, carried out by governments, whether it would be better to use targeted fortification for specific groups at risk of shortages instead of mass fortification (especially in terms of possible excess consumption in groups without deficiencies and the cost of the fortifier used). In countries where there is a high risk of nutrient deficiencies and the development of clinical symptoms, it is reasonable for governments to develop laws on food fortification, including bread flour, based on the health benefits for consumers documented in clinical trials. 

Some authors described previous work that suggests reasons that may be responsible for the changes caused by the fortifiers used. The reduction in TG levels can be attributed to the non-synergistic relationship between the K content of bread, changes in postprandial serum K levels, and the capacity of Mg and K to enhance glucose and insulin sensitivity [14].

Vitamin C potentially reduces gluten’s emulsifying properties and stability, improving dough viscosity by modifying sulfur bonds, obstructing carbohydrate breakdown, and lowering the glucose index [27].

Chromium(III) (Cr(III)) plays a role in regulating carbohydrate and lipid metabolism, and mediating ghrelin reduction, thereby enhancing insulin sensitivity, ameliorating hyperglycemia, suppressing free radical formation, and reducing SBP [21,22].

Several authors suggest that the significant attenuation of insulin may be attributed to the presence of fiber, pectin, and polyphenol compounds, which can reduce the rate and degree of starch digestibility [21,22,28,36,39]. Furthermore, arabinoxylan fibers may reduce the postprandial glucose response by absorbing water, altering the bread matrix structure, modifying starch granule accessibility to amylase enzymes, and increasing gastrointestinal tract content’s viscosity [29,31].

Additionally, soy consumption, with its high phytoestrogen isoflavone content, soluble fiber, and polyunsaturated fats, has hypocholesterolemic effects, reducing total cholesterol levels in the blood, which may be a result of phytoestrogen isoflavones (Fouad, Sharifi). Similar mechanisms are considered for using phytochemicals, such as flavonoids, in reducing systolic blood pressure [35].

Consumption of bread fortified with physiological compounds has revealed a positive correlation between plasma protein biosensors of inflammation (Apo J) and certain pathological conditions, including atherosclerosis and obesity indices [36].

## 5. Strength

According to our knowledge, this is the first systematic review of the health benefits of fortified bread confirmed with randomized clinical studies. This review was elaborated on according to the PRISMA model by two independent researchers who screened and assessed the internal quality of selected reports. The findings of this review involve randomized clinical studies and thus should be considered more reliable and assumed to be “gold standards” in measuring the efficacy of an intervention. Moreover, the Jadad scale used in this review offers an objective framework, eliminating subjectiveness.

## 6. Conclusions

This review provides valuable insights that substantially affect health policy and bread production practices. The introduction of mandatory fortification for bread emerges as a critical intervention in improving public health, especially in regions grappling with mineral, nutritional, and vitamin deficiencies. By fortifying commonly consumed bread, the population can gain enhanced access to these vital components, significantly contributing to achieving several Sustainable Development Goals such as Zero Hunger, as fortifying bread elevates its nutritional content, transforming it into a more substantive source of essential nutrients, thereby contributing to food security and combatting malnutrition. Thus, fortification of such commonly consumed products can be a tool leading to sustainable health. 

In our opinion, in future development, bread fortification must be applied prudently to achieve effective programs according to responsible policies, including rational marketing of fortified products. Furthermore, advancements in nutritional technology should be directed towards developing suitable forms of fortifiers utilized for bread enrichment, ensuring their bioavailability is preserved during the baking process. Fortification effects must be monitored diligently, and the public or customers should be informed effectively about its benefits through educational activities by the government or producers. Moreover, establishing transparent fortification guidelines should be developed according to the changing demographics of the global population and, thus, changes in the food supply or advances in functional food technology.

Partnerships for the Goals, as collaborative endeavors between health policy makers, bread producers, and other stakeholders, become pivotal in successfully implementing mandatory fortification and ensuring the widespread availability of nutrient-rich bread. Such partnerships hold the potential to drive sustainable advancements in public health and nutrition, foster societal change, and accelerate progress towards achieving sustainable health. 

However, in light of inadequate nutrition education, caution should be exercised in bread fortification, refraining from incorporating substances that may lead to adverse effects due to excessive intake of macro- or microelements.

It is important to acknowledge that synthetic fortifiers, specifically those composed of vitamins, have the potential to exert xenobiotic-like effects on the human body, potentially leading to disruptions in liver and kidney metabolism. Consequently, the precise form and quantity of fortifications employed bear considerable significance.

Based on the aforementioned clinical trial outcomes analysis, incorporating fortified bread into nutritional preventive strategies and dietary interventions for specific disorders could yield favorable health outcomes.

## Data Availability

The bibliographic query in Repository for Open Data, https://doi.org/10.18150/FTZ4JI (accessed on 28 March 2023).
